# Exploring the molecular landscape of osteosarcoma through PTTG family genes using a detailed multi-level methodology

**DOI:** 10.3389/fgene.2024.1431668

**Published:** 2024-07-30

**Authors:** Yulin Lu, Danjun Wang, Guoao Chen, Zitong Shan, Dongmei Li

**Affiliations:** ^1^ School of Medicine, Shihezi University, Shihezi, Xinjiang, China; ^2^ Key Laboratory of Xinjiang Endemic and Ethnic Diseases, School of Medicine, Shihezi University, Shihezi, Xinjiang, China; ^3^ NHC Key Laboratory of Prevention and Treatment of Central Asia High Incidence Diseases, School of Medicine, Shihezi University, Shihezi, Xinjiang, China

**Keywords:** osteosarcoma, PTTG family genes, biomarker, prognosis, molecular landscape

## Abstract

**Background:**

Osteosarcoma (OS) poses a significant clinical challenge, necessitating a comprehensive exploration of its molecular underpinnings.

**Methods:**

This study explored the roles of PTTG family genes (PTTG1, PTTG2, and PTTG3P) in OS, employing a multifaceted approach encompassing molecular experiments, including OS cell lines culturing, RT-qPCR, bisulfite and Whole Exome Sequencing (WES) and *in silico* experiments, including The Cancer Genome Atlas (TCGA) and Gene Expression Omnibus (GEO) datasets-based validation, overall survival, gene enrichment, functional assays, and molecular docking analyses.

**Results:**

Our findings reveal a consistent up-regulation of PTTG genes in OS cell lines, supported by RT-qPCR experiments and corroborated across various publically available expression datasets databases. Importantly, ROC curve analyses highlight their potential as diagnostic markers. Moving beyond expression profiles, we unveil the epigenetic landscape by demonstrating significant hypomethylation of CpG islands associated with PTTG genes in OS. The negative correlation between methylation status and mRNA expression emphasizes the regulatory role of promoter methylation in PTTG gene expression. Contrary to expectations, genetic mutations in PTTG genes are rare in OS, with only benign mutations observed. Moreover, functional assays also confirmed the oncogenic roles of the PTTG gene in the development of OS. Lastly, we also revealed that Calcitriol is the most appropriate drug that can be utilized to treat OS in the context of PTTG genes.

**Conclusion:**

The identification of PTTG genes as potential diagnostic markers and their association with epigenetic alterations opens new avenues for understanding OS pathogenesis and developing targeted therapies. As we navigate the complex landscape of OS, this study contributes essential insights that may pave the way for improved diagnostic and therapeutic strategies in its management.

## Introduction

Osteosarcoma (OS), characterized by the malignant proliferation of osteoblasts, stands as the most common primary bone sarcoma, predominantly affecting adolescents and young adults (Zhao et al., 2021). This aggressive malignancy poses substantial challenges in both diagnosis and treatment, warranting a comprehensive exploration of its intricate landscape (Beird et al., 2022). Already reported notable biomarkers of OS include P-glycoprotein (P-gp), multidrug resistance protein 1 (MRP1), and osteopontin (OPN) (Kang, 2023). While these biomarkers have shown promise, they come with limitations. For instance, P-gp and MRP1 are associated with chemoresistance, posing challenges in treatment efficacy (Binkhathlan and Lavasanifar, 2013; Patel et al., 2024). Additionally, OPN, although linked to OS progression, lacks specificity and sensitivity for accurate diagnosis and prognosis prediction (Zhang et al., 2020). Moreover, biomarkers like alkaline phosphatase (ALP) and lactate dehydrogenase (LDH), while commonly used in clinical practice, lack specificity for OS, as elevated levels can be indicative of various other conditions (Jiang et al., 2023). Moreover, despite notable progress in adjuvant chemotherapy (Luo et al., 2023), the formidable migratory and invasive characteristics of OS thwart endeavors to mitigate its mortality rate (Onciul et al., 2024). The elevated mortality rate is linked to the limited research on the mechanisms underlying metastatic OS. Comprehending the intricacies of OS metastasis development is pivotal, not only for defining crucial diagnostic or prognostic biomarkers but also for informing the clinical management of OS. The understanding of these molecular pathways offers the potential for identifying targeted therapeutic interventions, thereby paving the way for precision medicine in OS.

The PTTG family, consisting of PTTG1, PTTG2, and PTTG3P, has been identified as a group of multifunctional genes with diverse roles in cellular processes, including cell cycle regulation, DNA repair, and apoptosis (Perramón and Jiménez, 2022). While initially recognized for its association with pituitary tumors, emerging evidence suggests that the PTTG family’s influence extends beyond the pituitary gland, encompassing various cancer types (Gong et al., 2022). A number of studies have demonstrated that PTTG1 is an oncogene, and is overexpressed in various cancers, including breast cancer, colorectal cancer, lung cancer, and kidney cancer (Li et al., 2013; Ren and Jin, 2017; Tian et al., 2022). PTTG2 and PTTG3P, identified as homologous genes of PTTG1, have recently garnered attention in the scientific community (Chen et al., 2000). Although their biological functions remain largely elusive, studies have unveiled their close association with the progression of various human cancer types. For instance, recent studies (Genkai et al., 2006; Guo et al., 2016; Grzechowiak et al., 2020) showcased a significant up-regulation of PTTG2 expression in glioblastoma, where its overexpression facilitated glioblastoma cell proliferation and invasion. Similarly, other studies (Weng et al., 2017; Zhou et al., 2019; Fei et al., 2022) illustrated the role of PTTG3P in enhancing the *in vitro* proliferation and invasion of gastric cancer cells, correlating its presence with poor prognosis. As of now, there has been no comprehensive examination of the expression patterns and prognostic implications of the PTTG family in OS.

The integration of *in silico* and molecular experimental approaches provides a powerful toolkit for unraveling the intricacies of cancer biology (Ahmad et al., 2022; Yasir et al., 2022; Xu et al., 2023; Huang et al., 2024). *In silico* analyses, such as bioinformatics and computational modeling offer a high-throughput and systematic exploration of large-scale genomic datasets (Ji et al., 2017; Usman et al., 2020; Usman and Hameed, 2023; Dong et al., 2024). These analyses can unveil potential associations, pathways, and molecular signatures that may guide subsequent experimental investigations. Molecular experiments, on the other hand, provide a closer look at the functional aspects of genes within the cellular context (Hong et al., 2024). This study was initiated to investigate the diagnostic and prognostic potential of PTTG family genes in OS using a comprehensive multilevel methodology. The outcomes of this study revealed significant up-regulation of PTTG1, PTTG2, and PTTG3P genes in OS. Moreover, promoter methylation analysis indicated hypomethylation of these genes in OS, suggesting their potential role in OS pathogenesis. Mutational analysis via Whole Exome Sequencing identified a benign mutation in PTTG1, with no mutations in PTTG2 and PTTG3P. Validation using The Cancer Genome Atlas (TCGA) and Gene Expression Omnibus (GEO) datasets confirmed the overexpression of these genes in OS. Survival analysis demonstrated that high mRNA expression of PTTG1, PTTG2, and PTTG3P was significantly associated with poor overall survival in OS patients. Functional analysis, including knockdown experiments, highlighted the oncogenic effects of these genes in OS. Moreover, drug prediction analysis identified Calcitriol as a potential therapeutic agent targeting PTTG1 and PTTG2. Overall, our findings shed light on the role of PTTG genes in OS progression and prognosis, paving the way for further research into their therapeutic potential and molecular mechanisms in OS.

## Methodology

### Cell culture

A total of 15 OS cell lines, including Saos-2, U-2 OS, MG-63, SJSA-1, HOS, 143B, KHOS/NP, MNNG/HOS, Cal 72, G-292, Saos-2/VP16, U-2 OS/RA, TE-85, TE-671, and Hs 895.T, and 5 control cell lines, including hFOB 1.19, HS 27, HUVEC, NHDF, NHOst were purchased from the American Type Culture Collection (ATCC). These cell lines were grown in appropriate mediums, including McCoy’s 5A Medium, Minimum Essential Medium (MEM), RPMI-1640 Medium, and Eagle’s Minimum Essential Medium (EMEM), supplemented with 4 mL L-glutamine and sodium pyruvate, along with 10% characterized fetal bovine serum (FBS). Cell line authenticity was confirmed using short tandem repeat (STR) analysis (Promega, Madison, WI, United States), while the absence of *mycoplasma* contamination was verified using the MycoAlert™ *mycoplasma* detection kit (Lonza, Switzerland).

### Nucleic acid extraction

The extraction of DNA from cell lines followed the organic method outlined in reference (Gupta, 2019), ensuring a systematic and established approach to preserve the genetic material. This method involves tissue homogenization to break down cellular structures, followed by cell lysis using detergents and proteases. Organic solvents such as phenol and chloroform are then used for DNA extraction, followed by precipitation and washing steps to purify the DNA. Concurrently, RNA isolation was conducted using the TRIzol method, following the detailed procedure outlined in reference (Rio et al., 2010). This method entails phase separation using TRIzol, a reagent designed to separate RNA, DNA, and proteins into distinct phases. Following phase separation, RNA precipitation was achieved by adding isopropanol, facilitating the isolation of RNA from the aqueous phase. Subsequent washing steps with 70% ethanol were performed to remove contaminants and impurities, ensuring the purity of the isolated RNA. Finally, the purified RNA was resuspended in an appropriate buffer, rendering it suitable for downstream applications.

### RT-qPCR-based expression analysis

cDNA was synthesized utilizing the cDNA Synthesis Mix Kit from Innovagene, located in Hunan, China. Real-time PCR was carried out employing the SYBR Green qPCR Mix also provided by Innovagene. The RT-qPCR assay was optimized using the serial dilution and standard curve methods outlined in a prior investigation (Taylor et al., 2015). The expression profiles of PTTG genes were calculated and analyzed using the 2^−ΔΔCq^ method. To ensure normalization, Glyceraldehyde-3-phosphate dehydrogenase (GADPH) was employed as an internal control or reference. The following primers were used for the amplification of GAPDH and PTTG family genes.

GAPDH-F 5′-ACC​CAC​TCC​TCC​ACC​TTT​GAC-3′,

GAPDH-R 5′-CTG​TTG​CTG​TAG​CCA​AAT​TCG-3'

PTTG1-F: 5′-GCT​TTG​GGA​ACT​GTC​AAC​AGA​GC-3′

PTTG1-R: 5′-CTG​GAT​AGG​CAT​CAT​CTG​AGG​C-3′

PTTG2-F: 5′-CTT​TGG​GCA​CTG​TCA​ACA​GAG​C-3′

PTTG2-R: 5′-TCT​GGA​TAG​GCG​TCA​TCT​GAG​G-3′

PTTG3P-F: 5′-CTG​CCT​GAA​GAG​CAC​CAG​ATT​G-3′

PTTG3P-R: 5′-CAT​GGT​GGA​GAG​GGC​ATC​TTC​A-3′

### Promoter methylation analysis

To construct normal BS-seq libraries, 10 μg of genomic DNA underwent fragmentation using a Covaris sonication system (Covaris S2). Following fragmentation, libraries were generated following the Illumina Paired-End protocol, which involved end repair, addition of < A > bases, and ligation of methylated adaptors. The ligated DNA then underwent bisulfite conversion using the EZ DNA Methylation-Gold kit (ZYMO) and was subsequently amplified via PCR. PCR was carried out in a final reaction volume of 50 μL, comprising 20 μL of purified DNA, 4 μL of 2.5 mM dNTP, 5 μL of 10X buffer, 0.5 μL of JumpStart™ Taq DNA polymerase, 2 μL of 10 uM PCR primers, and 37.5 μL of water. The thermal cycling program included an “initial step at 94°C for 30 s, followed by 10 cycles of 94°C for 30 s, 60°C for 30 s, and 72°C for 30 s, with a final extension at 72°C for 1 min” Subsequent sequencing was performed using the HighSeq2000 platform (Illumina). Methylation levels were normalized as beta values.

### Receiver operating characteristic (ROC) curve

ROC curves were employed to assess the discriminative capability of PTTG family genes in identifying OS. The area under the ROC curves, along with 95% confidence intervals was calculated. This index was utilized to ascertain the optimal cut-off value. Sensitivity, specificity, positive predictive values (PPV), and negative predictive values (NPV) were computed to further characterize the performance of the ROC curves. Sensitivity (calculated as the ratio of true positive results to the total number of actual positive cases) represents the proportion of true positive results among individuals with the condition of interest, while specificity (calculated as the ratio of true negative results to the total number of actual negative cases) indicates the proportion of true negative results among individuals without the condition. PPV reflects the proportion of true positive results among individuals who tested positive for the condition, whereas NPV denotes the proportion of true negative results among individuals who tested negative for the condition. The ROC curve analysis was carried out using Graph Pad Prism 7.0 with data derived from RT-qPCR and methylation analysis.

### Mutational analysis of the PTTG genes

The exploration of mutations within the PTTG family genes was conducted using the Whole Exome Sequencing (WES) method. Ten OS samples were sent to the Beijing Genomics Institute (BGI), where WES was executed following a detailed protocol:

Genomic DNA extracted from the 10 OS samples served as the foundation for creating targeted capture pulldown and exon-wide libraries. This process involved utilizing the xGen^®^ Exome Research Panel from Integrated DNA Technologies, Inc., based in Illinois, United States, and the TruePrep DNA Library Prep Kit V2 for Illumina (#TD501, Vazyme, Nanjing, China). The resultant captured libraries underwent pair-end sequencing on the Illumina HiSeq 2500 platform. Following sequencing, the reads were meticulously processed and aligned to the GRCh37/hg19 human genome reference assembly, with an emphasis on identifying germline variations. Local rearrangements were implemented to enhance the alignment of individual reads. The identification of SNPs and insertion–deletion (indel) variants was carried out using the GATK’s Best Practices Workflow, as outlined in detail at https://github.com/Sydney-Informatics-Hub/Somatic-ShortV. To comprehensively understand these procedures, HaplotypeCaller was employed for detecting germline short variants, while Mutech2 caller was utilized to identify somatic short variants, encompassing SNVs and indels. The observed genetic mutations were then interpreted in accordance with the “American College of Medical Genetics and Genomic (ACMG)” guidelines (Richards et al., 2015) and annotated using the ClinVar database (Landrum et al., 2014).

### Validation of PTTG gene expression using TCGA and GEO datasets

UALCAN (https://ualcan.path.uab.edu/index.html) is a user-friendly, web-based platform that facilitates the exploration of cancer transcriptome data (Chandrashekar et al., 2017). Leveraging The Cancer Genome Atlas (TCGA) database, UALCAN enables users to analyze gene expression and survival data across various cancer types. In the present study, we used the UALCAN database for the validation of PTTG family gene expression at the mRNA level across OS TCGA datasets.

We acquired the standardized matrix profile (*series matrix.txt) of GSE12865, a microarray dataset, and extracted patient information from the Gene Expression Omnibus (GEO, https://www.ncbi.nlm.nih.gov/geo/) database (Clough and Barrett, 2016). The dataset’s platform is the GPL10295 Illumina human-6 v2.0 expression beadchip. To ensure consistency, we excluded probes not aligned with the Gene symbol using the platform annotation file. In cases where different probes corresponded to the same gene, we calculated their median expression values to obtain the final gene expression value. Utilizing the “limma” (linear models for microarray data) R package, we discerned the expression levels of PTTG family genes between OS and control samples, applying criteria of False Discovery Rate (FDR) < 0.05 and an absolute log2fold change (FC) > 1.

### Validation of PTTG gene promoter methylation level and mutational analysis across TCGA datasets

MEXPRESS (http://mexpress.be) is a versatile online tool for visualizing and analyzing DNA methylation data from The Cancer Genome Atlas (TCGA). This user-friendly platform offers interactive visualizations of DNA methylation patterns across various genes, allowing researchers to explore correlations with clinical parameters (Tang et al., 2022). In this study, we used MEXPRESS database to verify the promoter methylation status of the PTTG family genes within TCGA OS patients.

cBioPortal (https://www.cbioportal.org/) is a dynamic web resource empowering researchers to explore and analyze multidimensional cancer genomics data (Cerami et al., 2012). Leveraging datasets from projects like TCGA and cBioPortal facilitates the visualization of genetic alterations, copy number variations, and clinical outcomes across diverse cancer types. For our investigation, we employed the cBioPortal database to conduct mutational analysis on PTTG family genes among patients with osteosarcoma from The Cancer Genome Atlas (TCGA) dataset.

### Survival analysis and constriction of PTTG gene-based prognostic model

The Kaplan Meier (KM) Plotter (https://kmplot.com/analysis/) is a powerful tool designed for survival analysis based on gene expression (Lánczky and Győrffy, 2021). With a user-friendly interface, the KM Plotter facilitates the exploration of biomarker significance, aiding researchers in identifying genes associated with prognosis. In our current study, we utilized the KM plotter tool to conduct a survival analysis focusing on PTTG family genes in patients with OS.

The predictive model was constructed through the utilization of Lasso and multivariate Cox proportional hazard regression analyses. This process was facilitated using the “survival” package within the R language. The TCGA-SARC dataset served as the training dataset, while the E_TABM_1202, GSE59455, GSE17679, and GSE16091 datasets were utilized for validation purposes. Positive coefficients identified in the analysis indicated an elevated risk of an event, such as death, while negative coefficients suggested a reduced risk. The magnitude of these coefficients reflected the impact of variables on hazard rates. This critical information was employed in the development of prognostic models for survival outcomes. The formula for the prognostic model in osteosarcoma patients was expressed as follows: the risk score equals the summation of the multivariate Cox regression coefficient variations of each mRNA.

### Enrichment analysis

DAVID tool (https://david.ncifcrf.gov/) is an invaluable bioinformatics tool aiding researchers in unraveling disease-related biological pathways (Sherman et al., 2022). By integrating diverse genomic resources, DAVID identifies enriched biological themes within gene datasets, facilitating functional annotation and pathway analysis. Its user-friendly interface streamlines the exploration of gene functional relationships, supporting researchers in comprehending the biological significance of their data. We performed the enrichment analysis of PTTG family genes using the DAVID.

### Exploration of PTTG expression regulatory drugs

DrugBank database (https://go.drugbank.com/) provides extensive information on drugs, drug targets, and drug interactions (Wishart et al., 2008). It serves as a valuable resource for researchers, clinicians, and pharmaceutical professionals, offering data on drug properties, mechanisms of action, and pharmacokinetics. In our investigation, we leveraged the resources provided by DrugBank to explore potential drugs capable of regulating the expression of PTTG family genes for the treatment of osteosarcoma.

### Knockdown of PTTG genes in OS cell line

siRNAs aimed at the knockdown of PTTG genes were acquired from OBiO Company. To silence PTTG family genes (PTTG1, PTTG2, and PTTG3P), the MG-63 cell lines underwent transfection with siRNA, utilizing a Transfection Reagent (INTERFERin, French).

### Cell counting Kit-8 and colony formation assays

Cell proliferation was assessed using the cell counting kit-8 (CCK-8) assay (Beyotime, Shanghai, China). Human MG-63 cells (1 × 10^5^) were plated in each well of a 96-well plate and cultured for 24, 48, and 72 h. Absorbance at 450 nm was measured using a Varioskan Flash spectrophotometer (Bio-Rad, United States). For the colony formation assay, 1 × 10^3^ cells were seeded in 6-well plates and incubated for 7 days in a culture medium supplemented with 10% FBS, allowing colony formation to occur.

### Molecular docking analysis

To evaluate the binding affinities between the selected drug, Calcitriol, and the PTTG1 and PTTG2 proteins, we utilized a molecular docking approach facilitated by the CB-DOCK web server (http://clab.labshare.cn/cb-dock/) (Liu et al., 2020). The SDF structure of Calcitriol was obtained from the PubChem database. PDB structures for the PTTG1 and PTTG2 proteins were generated using the SwissModel tool (https://swissmodel.expasy.org/). The process involved several crucial steps, including pre-processing of the ligand (Calcitriol), removal of excess ligands from the target proteins (macromolecules), elimination of crystal water molecules, and addition of hydrogen atoms. Subsequently, molecular docking was conducted using the CB-DOCK platform to compute the binding energies of the molecules across various conformations. Binding energies falling within the range of −5 kcal/mol to −10 kcal/mol or lower were considered favorable. The conformation exhibiting the highest hydrogen bond energy was chosen as the active component for protein interaction analysis. For visualization purposes, PYMOL software (version 2.5.2) was employed.

## Statistics

Meticulous statistical analyses were conducted to ensure the robustness of our data analysis. For Gene Ontology (GO) and Kyoto Encyclopedia of Genes and Genomes (KEGG) enrichment analysis, Fisher’s Exact test was employed to compute the differences in gene sets, a widely recognized method for identifying significant biological pathways and molecular functions associated with dysregulated genes in OS. This statistical approach enabled us to precisely pinpoint the relevant pathways and functions implicated in OS pathogenesis.

Furthermore, correlational analyses were performed using the Pearson correlation method, allowing for a comprehensive understanding of the intricate relationship between these molecular events in OS progression. Additionally, comparisons between different experimental groups were made using the Student’s t-test, providing statistical significance to our findings by assessing the differences between groups with rigorous statistical measures. A *p*-value < 0.05 was considered significant.

All statistical analyses were executed using R version 3.6.3 software, a widely utilized platform renowned for its versatility and reliability in statistical computing and data analysis, ensuring the validity and reproducibility of our results.

## Results

### Expression of PTTG family genes in OS and control cell lines

**FIGURE 1:**
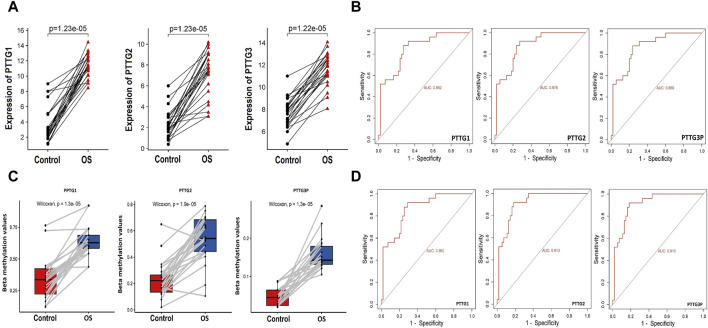
Expression, promoter methylation, and ROC analysis of the PTTG genes. **(A)** RT-qPCR-based relative expression levels of the PTTG genes in OS cell lines compared to control cell lines determined. **(B)** RT-qPCR expression-based Receiver Operating Characteristic (ROC) analysis illustrating the diagnostic accuracy of PTTG gene expression in distinguishing OS from control samples. **(C)** Bisulfite sequencing-based relative promoter methylation levels of the PTTG gene in OS cell lines versus control cell lines assessed through bisulfite sequencing. **(D)** Promoter methylation level-based ROC analysis evaluating the diagnostic utility of PTTG gene promoter methylation levels in distinguishing OS samples from controls. A significance threshold of *p* < 0.05 was applied.

The RT-qPCR experiment assessed the mRNA expression levels of PTTG1, PTTG2, and PTTG3P genes in 15 OS cell lines and 5 control noncancerous cell lines. Notable differences in the expression of these three genes were observed between OS and control cell lines. Each gene exhibited a consistent up-regulation tendency in OS cell lines compared to the controls, as illustrated in Figure 1A. This up-regulation was statistically significant, indicating the potential role of PTTG1, PTTG2, and PTTG3P as biomarkers for OS. ROC curves were generated to assess the diagnostic capability of the up-regulated PTTG1, PTTG2, and PTTG3P genes in distinguishing between OS and normal individuals. The curves demonstrated that these genes possess the capability to effectively differentiate between OS and control samples (Figure 1B). The area under the curve (AUC) values indicated high diagnostic accuracy, suggesting the utility of PTTG1, PTTG2, and PTTG3P as potential biomarkers for OS detection and prognosis.

### Promoter methylation levels of PTTG family genes in OS and control cell lines

Studying promoter methylation levels is crucial as it serves as a causative factor in gene expression dysregulation. Alterations in methylation patterns can silence or activate gene expression, impacting cellular processes. In the current work, the bisulfite sequencing technique was employed to investigate whether the high expression of PTTG1, PTTG2, and PTTG3P genes is linked to promoter methylation in OS. The findings revealed a significant reduction in the methylation levels of the PTTG1, PTTG2, and PTTG3P promoters in OS cell lines (n = 15) compared to control cell lines (n = 05), as depicted in Figure 1C. This suggests that PTTG1, PTTG2, and PTTG3P genes undergo hypomethylation in OS cell lines compared to the controls. Additionally, ROC curves demonstrated that the hypomethylation of PTTG1, PTTG2, and PTTG3P serves as a marker for distinguishing between OS and control samples (Figure 1D). These findings suggest a potential role for promoter hypomethylation of PTTG1, PTTG2, and PTTG3P genes in the pathogenesis of osteosarcoma. They underscore the diagnostic utility of these genes as biomarkers for this malignancy.

### Mutational landscape of PTTG family genes across OS cell lines

As indicated in a previous report, genetic mutations are recognized as significant contributors to the onset and progression of OS (Rickel et al., 2017). To investigate the genomic changes in PTTG1, PTTG2, and PTTG3P within OS, we conducted WES analysis on ten OS cell lines. Interestingly, our analysis revealed only a single benign mutation NM_004219.4(PTTG1).5C>T (p.Ala2Val)< in PTTG1 gene. No mutations were identified in the other two genes, namely, PTTG2 and PTTG3P. The identified mutation in PTTG1 [NM_004219.4(PTTG1).5C>T (p.Ala2Val)] represents a benign alteration. While this mutation does not confer a pathogenic effect, its presence suggests genetic variability within the PTTG1 gene in OS. Further functional studies may elucidate the impact of this mutation on PTTG1 protein function and its role in OS development and progression.

### Validation of PTTG genes expression using TCGA and GEO datasets

**FIGURE 2:**
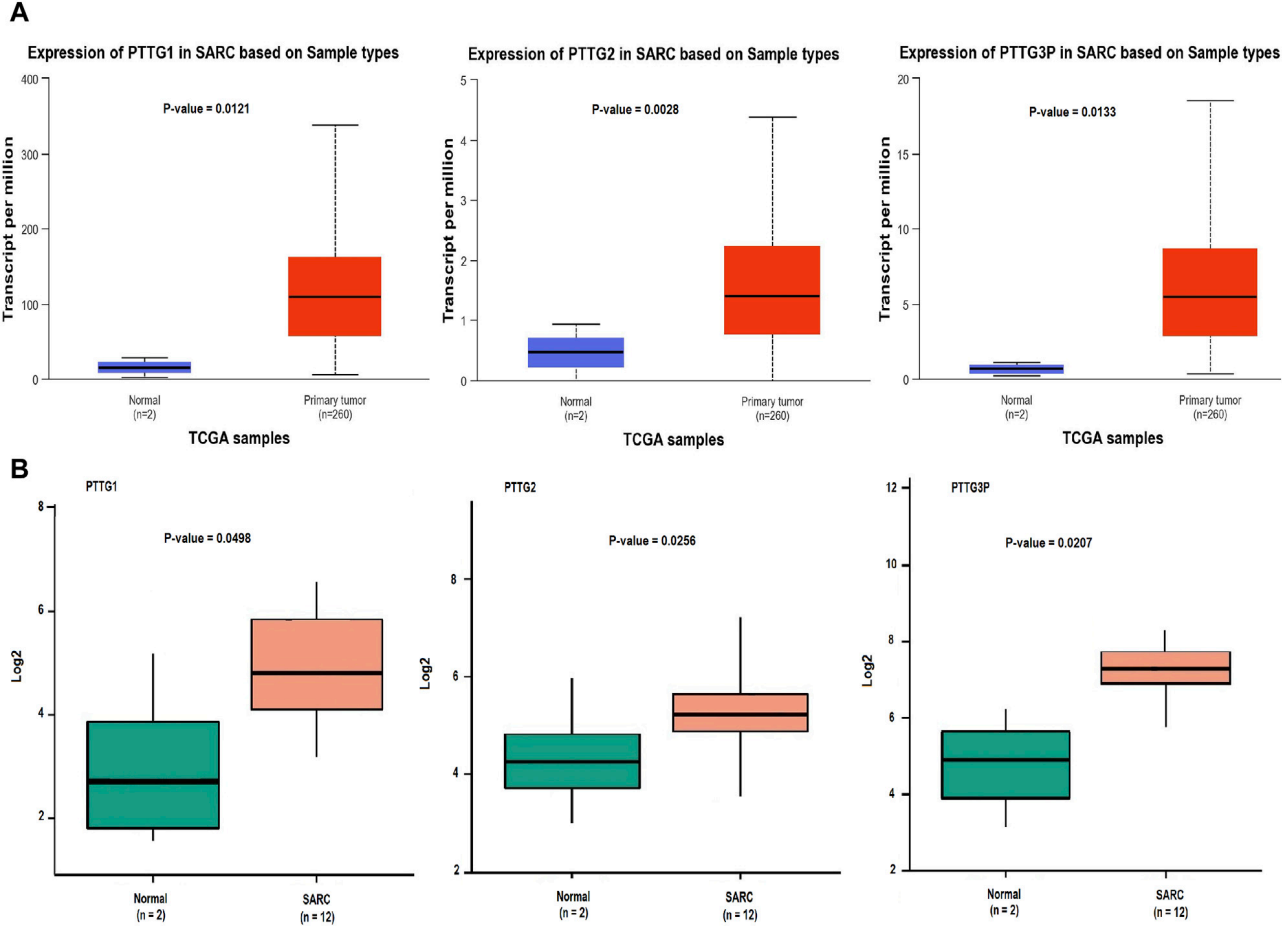
mRNA expression profiling of the PTTG genes across TCGA and GEO datasets. **(A)** Box plot presentation illustrating mRNA expression levels of PTTG genes in TCGA OS and normal control samples. **(B)** Box plot presentation depicting mRNA expression levels of PTTG genes in GEO OS and normal control samples. A significance threshold of *p* < 0.05 was applied.

Initially, we referenced the UALCAN database to validate the expression patterns of PTTG1, PTTG2, and PTTG3P genes. Notably, in comparison to the control group (n = 02), the OS group (n = 200) exhibited markedly elevated mRNA levels of PTTG1, PTTG2, and PTTG3P, as illustrated in Figure 2A.

Subsequently, we used the GEO database to examine the expression of PTTG1, PTTG2, and PTTG3P genes. The mRNA levels of PTTG1, PTTG2, and PTTG3P were notably up-regulated in the OS group (n = 02) as opposed to the control group (n = 12), as depicted in Figure 2B. These outcomes aligned with the results obtained from the RT-qPCR analysis. Collectively, our results from both the UALCAN and GEO databases, in conjunction with RT-qPCR analysis, provide compelling evidence of the aberrant expression of PTTG1, PTTG2, and PTTG3P genes in OS. These findings emphasize the potential significance of these genes in the pathogenesis of OS.

### Validation of PTTG gene promoter methylation level and mutational analysis across TCGA datasets

**FIGURE 3:**
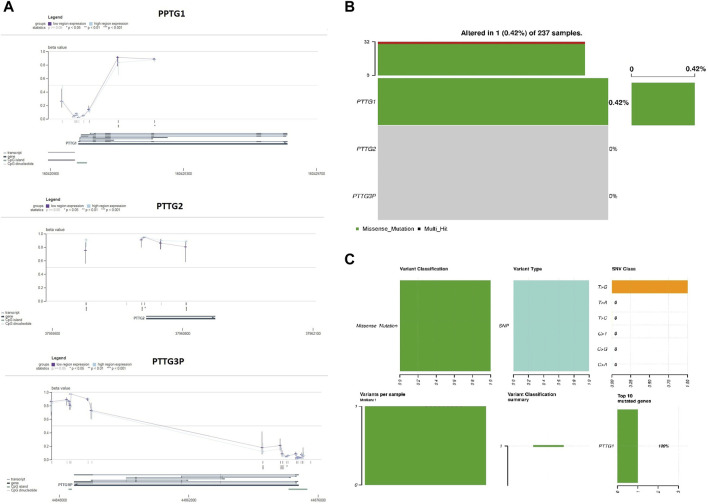
Validation of promoter methylation levels and mutational analysis of PTTG genes in TCGA OS samples using the MEXPRESS and cBioportal databases. **(A)** Correlations of PTTG genes prompter methylation levels with their expression levels via the MEXPRESS database. **(B)** The proportion of OS samples exhibiting mutations in PTTG genes sourced from the cBioPortal database. **(C)** Summary of observed genetic alterations in PTTG genes across OS samples. A significance threshold of *p* < 0.05 was applied.

Next, we utilized the MEXPRESS data visualization tool to present DNA methylation and expression data for PTTG1, PTTG2, and PTTG3P in OS. According to Pearson’s correlation coefficient, there was a negative correlation between the mRNA expression of PTTG1, PTTG2, and PTTG3P and the degree of methylation of the CpG islands (Figure 3A). These findings further affirm the significant role of promoter methylation in regulating the expression of PTTG1, PTTG2, and PTTG3P.

Additional scrutiny using the cBioPortal tool identified a single gene mutation in the nucleotide sequence of PTTG1 (Figures 3B, C). Importantly, the identified mutation was of a benign nature. In summary, the conjecture arises that genetic mutations are infrequent in PTTG1, PTTG2, and PTTG3P genes among OS patients. Overall, our findings validate that promoter methylation plays a crucial role in regulating the expression of PTTG1, PTTG2, and PTTG3P in OS, while genetic mutations within these genes are rare.

### Prognostic model development related to PTTG family genes

**FIGURE 4:**
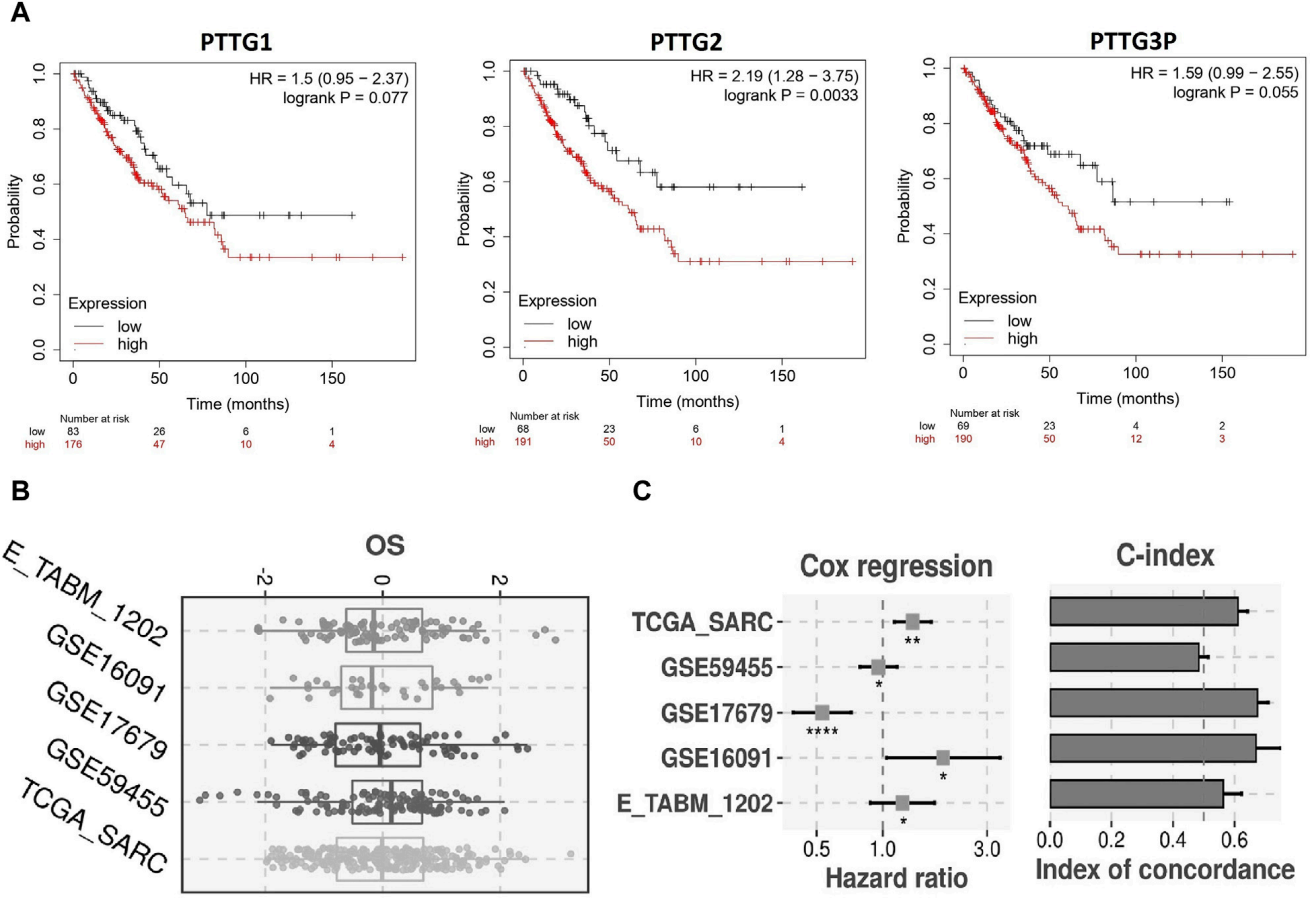
Survival analysis and construction of the PTTG genes-based prognostic model. **(A)** Kaplan-Meier (KM) plotter-based survival analysis of PTTG genes in TCGA OS samples. **(B)** Univariate Cox regression analysis evaluating the prognostic significance of PTTG genes. **(C)** Construction of risk scores based on PTTG gene expression. A significance threshold of *p* < 0.05 was applied.

Utilizing the best cut-off value (median), our analysis of 249 OS patients via the Kaplan–Meier plotter unveiled a significant correlation between elevated mRNA expression levels of PTTG1, PTTG2, and PTTG3P and diminished Overall Survival rates (Figure 4A). Subsequently, employing the TCGA-SARC dataset as the training dataset and E_TABM_1202, GSE59455, GSE17679, and GSE16091 datasets as validation sets, we constructed a prognostic model based on PTTG1, PTTG2, and PTTG3P genes. Employing a stepwise Cox regression model incorporating hazard ratio, c-index, and risk score parameters, our model effectively assessed OS patient prognosis across all analyzed datasets, as evidenced by robust evaluation via the c-index (Figure 4B). These findings emphasize the potential utility of our prognostic model in accurately predicting survival outcomes for OS patients (Figure 4C).

### Enrichment analysis of PTTG1, PTTG2, and PTTG3P genes reveals key GO and KEGG pathways

**FIGURE 5:**
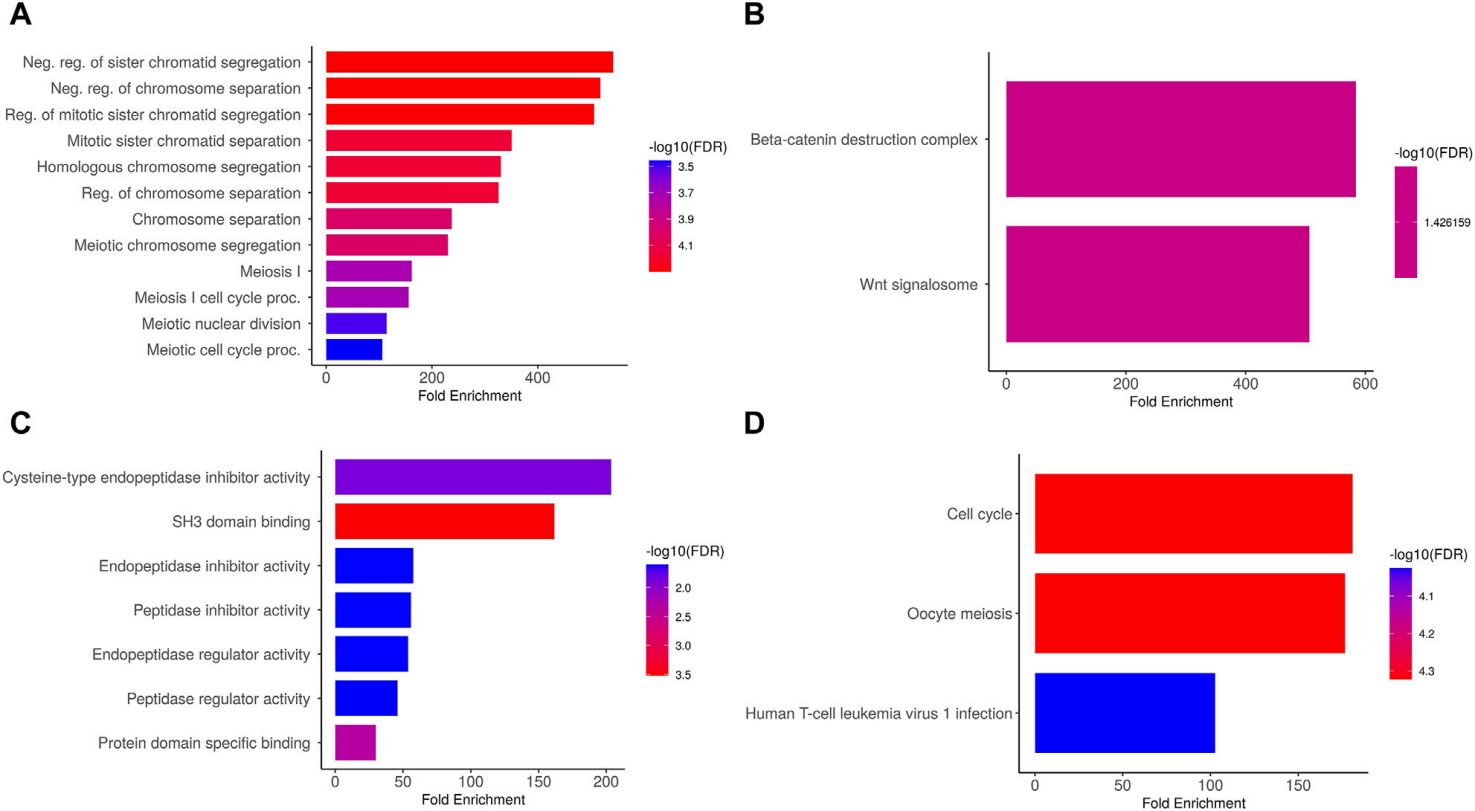
Gene enrichment analysis of PTTG genes via DAVID tool. **(A)** Cellular Component (CC), **(B)** Biological Process (BP), **(C)** Molecular Function (MF), and **(D)** Kyoto Encyclopedia of Genes and Genomes (KEGG) enrichment analysis of PTTG genes. A significance threshold of *p* < 0.05 was applied.

We conducted an analysis on the PTTG1, PTTG2, and PTTG3P genes to elucidate their GO and KEGG pathways in OS. In the CC, “Neg. reg of sister chromatid segregation, Neg. reg of chromosome separation, and Reg. of mitotic sister chromatid segregation, etc., terms were significantly associated with the PTTG1, PTTG2, and PTTG3P (Figure 5A).” Concerning MF, the “Beta-catenin destruction complex and Wnt signalosome terms were closely associated with the PTTG1, PTTG2, and PTTG3P (Figure 5B).” In BP, some vital functions including “Cysteine-type endopeptidase inhibitor activity, SH3 domain binding, and endopeptidase inhibitor activity, etc., terms were significantly associated with the PTTG1, PTTG2, and PTTG3P (Figure 5C).” Moreover, PTTG1, PTTG2, and PTTG3P-enriched KEGG pathways include “Cell cycle, Oocyte meiosis, and Human T-cell leukemia virus 1 infection, etc., (Figure 5D).” Overall, these findings provide valuable insights into the functional significance of PTTG1, PTTG2, and PTTG3P in OS.

#### Cell counting Kit-8 and colony formation assays

**FIGURE 6:**
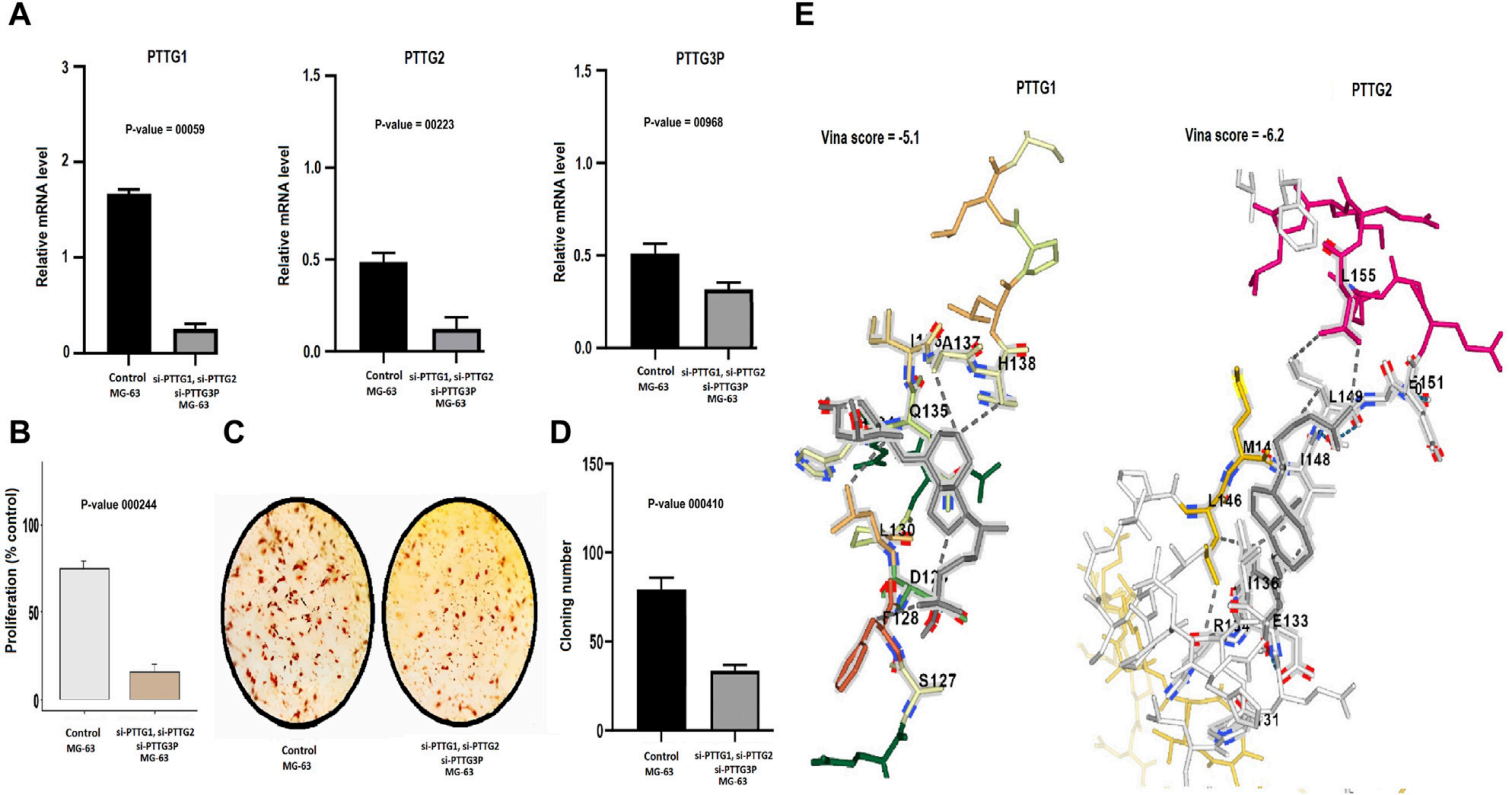
Knockdown of PTTG1, PTTG3, and PTTG3P impairs the growth and metastatic potential of MG-63 cells and conducting molecular docking analysis. **(A)** Assessment of siRNA transfection efficiency targeting PTTG1, PTTG2, and PTTG3P via RT-qPCR. **(B)** Analysis of proliferation in MG-63 cells post-transfection. **(C, D)** Colony formation assay depicting the impact of PTTG gene knockdown on colony formation ability. **(E)** Molecular docking simulation illustrating the interaction between Calcitriol and PTTG1/PTTG3 proteins. PTTG1 and PTTG3 proteins are represented in blue structures, while Calcitriol is depicted in gray molecules, demonstrating their docking interactions. A significance threshold of *p* < 0.05 was applied.

Simultaneous silencing of the PTTG1, PTTG2, and PTTG3P genes was performed in same MG-63 cells using siRNA to investigate their combined effects on key cellular processes such as cell cycle regulation, tumor growth, and other critical cellular functions. The silencing efficiency was checked with the help of RT-qPCR. The results, as depicted in Figure 6A, unequivocally demonstrated a significant reduction in the expression levels of PTTG1, PTTG2, and PTTG3P in the transfected MG-63 cells in comparison to the control MG-63 cells. To gain deeper insights into the repercussions of PTTG1, PTTG2, and PTTG3P knockdown, we conducted CCK-8 and colony-forming assays, providing compelling evidence of decreased cellular proliferation in the cells with silenced PTTG1, PTTG2, and PTTG3P, in contrast to the control A2058 cells (Figures 6B–D). These findings emphasize the importance of PTTG1, PTTG2, and PTTG3P in regulating cellular proliferation and suggest their potential as therapeutic targets in osteosarcoma.

### Prediction and molecular docking of the drugs related to PTTG family genes

Initially, DrugBank was utilized to predict drugs associated with PTTG1 and PTTG2 in the context of OS treatment. Within this database, one notable drug, Calcitriol, emerged as a significant candidate targeting PTTG1 and PTTG2 genes. The criteria employed to identify Calcitriol’s significance from DrugBank encompass following key aspects. Firstly, Calcitriol exhibited a capacity to target PTTG1 and PTTG2 genes, thereby reducing their expression. Secondly, its pharmacological attributes, including mechanism of action and safety profile as documented in DrugBank, render it a viable option for OS therapy. To further validate the role of Calcitriol in reducing expression, molecular docking analysis was conducted. The docking results revealed that Calcitriol exhibited varying binding affinities with PTTG1 and PTTG2, ranging from −5.1 to −6.2 kcal/mol (Figure 6E). The binding affinities, ranging from −5.1 to −6.2 kcal/mol, suggest a notably strong interaction between Calcitriol and the PTTG1 and PTTG2 proteins (Figure 6E). Overall, our findings highlight Calcitriol as a promising candidate for further preclinical and clinical investigations in OS treatment.

## Discussion

OS stands as a formidable challenge in the realm of oncology, particularly due to its aggressive nature and limited treatment options for advanced cases (Eaton et al., 2021; Olatunji et al., 2023). While the role of PTTG has been established in various cancers, its specific functions and significance in OS remain less understood. Our study endeavors to address this gap by providing insights into the molecular pathways and biological processes associated with PTTG1, PTTG2, and PTTG3P in OS. By elucidating their roles in cell cycle regulation, signaling pathways, and tumor progression, we aim to shed light on potential therapeutic targets and diagnostic biomarkers for OS.

In the current study, we identified and validated the overexpression of the PTTG1, PTTG2, and PTTG3P genes in OS patients. We also observed that the overexpression of these genes was significantly associated with the poor overall survival of the OS patients. Furthermore, it was explored that the PTTG1, PTTG2, and PTTG3P genes were hypomethylated and slightly mutated in OS patients. Functional analysis, involving the knockdown of PTTG1, PTTG2, and PTTG3P, revealed the oncogenic effects of these genes in OS.

The gene enrichment analysis conducted on the PTTG1, PTTG2, and PTTG3P genes provided valuable insights into their functional roles and potential pathways in OS. In terms of CC, our analysis revealed significant associations of terms related to negative regulation of “sister chromatid segregation, negative regulation of chromosome separation, and regulation of mitotic sister chromatid segregation” with the PTTG1, PTTG2, and PTTG3P genes. These findings suggest the potential involvement of these genes in the control of chromosome dynamics during cell division, which could have implications for tumor progression and proliferation. Regarding MF, our results highlighted associations of the PTTG1, PTTG2, and PTTG3P genes with terms related to the “Beta-catenin destruction complex and Wnt signalosome.” This indicates their potential involvement in the regulation of signaling pathways crucial for cell proliferation, differentiation, and tumor development, particularly through interactions with the Wnt signaling pathway. In terms of BP, our analysis revealed significant associations of the PTTG1, PTTG2, and PTTG3P genes with functions such as “cysteine-type endopeptidase inhibitor activity, SH3 domain binding, and endopeptidase inhibitor activity.” These findings suggest potential roles for these genes in the regulation of “proteolytic processes, protein-protein interactions, and cellular signaling pathways” implicated in cancer progression and metastasis. Moreover, our study identified enriched KEGG pathways associated with PTTG1, PTTG2, and PTTG3P, including the “cell cycle, oocyte meiosis, and human T-cell leukemia virus 1 infection pathways.” These pathways are known to play critical roles in regulating cell division, DNA repair, and immune response, highlighting the multifaceted roles of these genes in OS pathogenesis.

The expression of PTTG1 showed a significant correlation with cell proliferation in laryngeal cancer patients (Ma et al., 2020). Remarkably, PTTG1 displayed heightened expression levels and showed an association with unfavorable prognosis among individuals diagnosed with kidney cancer (Wondergem et al., 2012; Gui et al., 2021). Via this study, we are the first to report overexpression and its association with poor overall survival of OS patients (as a prognostic factor) for the PTTG1, PTTG2, and PTTG3P genes. Previous studies indicate that PTTG2 contributes to the initiation of tumors (Méndez-Vidal et al., 2013; Liang et al., 2024). For instance, PTTG2 overexpression was found to be associated with enhanced cell proliferation in cancer patients (Guo et al., 2016). In the current investigation, a substantial overexpression of PTTG2 was observed in OS samples. Notably, this overexpression of PTTG2 was significantly associated with diminished overall survival in OS patients, establishing it as a prognostic factor for shorter overall survival. The upregulation of PTTG3P has been demonstrated to promote tumorigenesis by enhancing PTTG1 expression in cancer patients (Huang et al., 2018). Previous research has also suggested increased expression of PTTG3P in breast cancer patients, with its elevated levels correlating with unfavorable prognosis (Lou et al., 2019). In our present investigation, heightened expression of PTTG3P was identified in OS samples, and this elevated PTTG3P expression significantly correlated with an unfavorable overall survival outcome in OS patients. This establishes PTTG3P as a prognostic factor for shorter overall survival in the context of OS.

Lastly, we explored that Calcitriol is a valuable drug that can be used to treat OS in the context of PTTG family genes. Calcitriol is the active form of vitamin D and is the emerging promising treatment option for cancer due to its multifaceted effects (Díaz et al., 2015; Yao et al., 2024). Research suggests its potential in inhibiting cancer cell proliferation, inducing apoptosis, and suppressing angiogenesis (Díaz et al., 2015). Calcitriol demonstrates immune-modulatory effects, contributing to the body’s defense against cancer (Xu et al., 2013; Ghaseminejad-Raeini et al., 2023). Calcitriol exerts its effects by binding to the vitamin D receptor (VDR) present in osteosarcoma cells, which in turn regulates the expression of various genes involved in cell proliferation, differentiation, and apoptosis. Studies have demonstrated that calcitriol can inhibit the proliferation of osteosarcoma cells and induce cell cycle arrest, promoting apoptosis and reducing tumor growth (Díaz et al., 2015; Yao et al., 2024). Additionally, calcitriol’s anti-inflammatory and immune-modulating properties may enhance its therapeutic efficacy by altering the tumor microenvironment, making it less conducive to cancer progression (Abu el Maaty and Wölfl, 2017). Its ability to target diverse pathways makes Calcitriol an intriguing candidate for cancer therapy, offering new avenues for treatment with minimal side effects.

The study demonstrates several strengths that contribute to its significance in understanding OS. Firstly, it employs a comprehensive approach by utilizing multiple techniques, which allows for a thorough exploration of the role of PTTG genes in OS development and progression. Secondly, the study benefits from the use of large datasets from different online sources, which validate the findings and enhance the robustness of the results. Additionally, the inclusion of functional validation through knockdown experiments provides mechanistic insights into the oncogenic effects of PTTG genes in OS. Lastly, the identification of Calcitriol as a potential therapeutic agent targeting PTTG1 and PTTG2 offers promising avenues for novel treatment strategies in OS.

Despite the comprehensive nature of our study, it is essential to acknowledge its limitations, particularly regarding the sample size. Firstly, while adequate for various analyses, the sample size may still restrict the generalizability of findings. Secondly, the focus on specific genes, including PTTG1, PTTG2, and PTTG3P, may overlook other genes associated with OS, potentially limiting the comprehensiveness of the study. Thirdly, while knockdown experiments provide evidence of oncogenic effects, further studies elucidating the precise molecular mechanisms underlying PTTG gene involvement in OS are warranted. Lastly, although *in silico* analyses are valuable, they rely on computational predictions and may not fully capture the complexity of biological systems.

Moving forward, several avenues for future research and clinical translation emerge from this study. Firstly, further functional studies are needed to delve deeper into the mechanisms by which PTTG genes contribute to OS, including their role in cell cycle regulation, apoptosis, and interactions with other signaling pathways. Secondly, translational studies investigating the clinical utility of PTTG genes as prognostic markers or therapeutic targets in OS patients are warranted. Additionally, exploration of the broader landscape of genes associated with OS could provide a more comprehensive understanding of the disease. Finally, continued exploration of Calcitriol and other potential therapeutic agents targeting PTTG genes could lead to the development of novel treatment strategies for OS.

## Conclusion

In conclusion, our study elucidates the significant role of PTTG1, PTTG2, and PTTG3P genes in OS pathogenesis, diagnosis, and prognosis. Through comprehensive analysis of gene expression, promoter methylation, mutational profiling, and survival outcomes, we have provided compelling evidence of the dysregulation of these genes in OS samples compared to the controls. The up-regulation of PTTG1, PTTG2, and PTTG3P genes in OS samples, accompanied by hypomethylation and limited genetic mutations, emphasizes their potential as key drivers of OS development and progression. Furthermore, our findings demonstrate the prognostic significance of PTTG1, PTTG2, and PTTG3P expression levels, with high expression correlating with poor overall survival in OS patients. The development of a prognostic model based on these genes further strengthens their utility in predicting patient outcomes and guiding clinical management decisions. Functional assays highlight the synergistic impact of PTTG1, PTTG2, and PTTG3P knockdown on cellular proliferation, emphasizing their potential as therapeutic targets in OS treatment. Additionally, drug prediction and molecular docking analysis identify Calcitriol as a promising candidate for down-regulating PTTG1 and PTTG2 expression, offering new avenues for targeted therapy in OS. Overall, our study provides comprehensive insights into the molecular mechanisms underlying osteosarcoma (OS) pathogenesis. Furthermore, it offers potential diagnostic biomarkers and therapeutic strategies aimed at improving patient outcomes in this aggressive bone malignancy.

## Data Availability

The original data presented in the study are publicly available. This data can be found here: The UALCAN database can be accessed at https://ualcan.path.uab.edu/. The Gene Expression Omnibus (GEO) hosts the datasets GSE12865, GSE59455, GSE17679, and GSE16091, which can be found at https://www.ncbi.nlm.nih.gov/geo/. The TCGA-SARC and E_TABM_1202 datasets are available at ArrayExpress (https://www.ebi.ac.uk/biostudies/arrayexpress). Additional resources include MEXPRESS (https://mexpress.ugent.be/), cBioPortal (https://www.cbioportal.org/), Kaplan Meier (KM) Plotter (https://kmplot.com/analysis/), DAVID tool (https://david.ncifcrf.gov/), DrugBank database (https://go.drugbank.com)/, PubChem database (https://pubchem.ncbi.nlm.nih.gov/), SwissModel tool (https://swissmodel.expasy.org/), and CB-DOCK tool (http://clab.labshare.cn/cb-dock/).
